# Updates on Geographical Dispersion of *Leishmania* Parasites Causing Cutaneous Affections in Algeria

**DOI:** 10.3390/pathogens10030267

**Published:** 2021-02-25

**Authors:** Arezki Izri, Amina Bendjaballah-Laliam, Denis Sereno, Mohammad Akhoundi

**Affiliations:** 1 Parasitology-Mycology Department, Avicenne Hospital, AP-HP, 93009 Bobigny, France; arezki.izri@aphp.fr; 2 Unité des Virus Émergents (UVE: Aix-Marseille Univ-IRD 190-Inserm 1207-IHU Méditerranée Infection), 13005 Marseille, France; 3 Etablissement Public Hospitalier de Hadjout, Tipaza 42200, Algeria; amina688@yahoo.fr; 4 MIVEGEC, Institut de Recherche pour le Développement, Montpellier University, 34394 Montpellier, France; denis.sereno@ird.fr; 5 InterTryp, Institut de Recherche pour le Développement, Montpellier University, 34398 Montpellier, France

**Keywords:** cutaneous leishmaniasis, Leishmania major, Leishmania tropica, Leishmania infantum, PCR–RFLP

## Abstract

Leishmaniases are neglected tropical diseases of public health concern in Algeria. To update the geographical distribution of *Leishmania* spp. causing cutaneous affection, we examined a set of Giemsa-stained smears prepared from skin lesions of the patients suspected to have cutaneous leishmaniasis (CL) in various geographical areas in Algeria. The identification of *Leishmania* parasites was performed using microscopy, conventional PCR, and PCR–RFLP (PCR-Restriction Fragment Length Polymorphism) targeting ITS1-rDNA. Among 32 smears provided from 27 suspected patients with cutaneous lesions, no trace of parasites was observed in the smear of three patients using microscopy and molecular approaches. Furthermore, four patients presented at least two lesions. PCR–RFLP confirmed the presence of *Leishmania* in 29 smears prepared from 24 patients. Two biopsies, negative after microscopic examination, were found positive by PCR. Of these 29 PCR positive smears (24 patients), 20 were identified using RFLP–PCR as *L. major*, two as *L. tropica*, and two as *L. infantum.* We found *L. major* infected patients from Ain skhouna, Biskra, El M’hir, Ghardaïa, M’Sila, and Saida, in agreement with previously reported cases. Furthermore, we highlighted for the first time, the identification of *L. major* in the patients from Bourkika, Bou Kremissa, Bou Saada Clef, Hajout, Maghnia, Médéa, Menaceur, Messad, Mostaghanem, Nador, Oran, and Sidi Okba. A phylogenetic reconstruction performed with sequences collected from the PCR products confirmed these identifications. Our data provide additional information on the geographical extension of CL caused by *L. tropica* and *L. infantum* in Algeria.

## 1. Introduction

Leishmaniases are vector-borne diseases caused by obligate protozoan parasites from the genus *Leishmania* (Trypanosomatida: Trypanosomatidae), and transmitted by the bite of infected female phlebotomine sandflies (Diptera: Psychodidae), whose hosts/reservoirs are animals such as canids, rodents, marsupials, hyraxes, or humans [1]. Epidemiological cycles of leishmaniases fall into two broad categories: the zoonotic forms of leishmaniases (ZL), where the primary reservoirs are wild or domestic mammals, and anthroponotic forms (AL) for which humans are the primary reservoirs. Two clinical presentations are distinguished: visceral (VL) and cutaneous (CL). Leishmaniases are endemic in large areas of the tropics, subtropics, and the Mediterranean basin. In 2018, 92 and 83 countries or territories were considered endemic or previously reported for CL and VL, respectively [2]. There are approximately 350 million people at risk for leishmaniases, and about 12 million infected cases worldwide, with an estimated annual incidence of 0.7–1.2 million for CL, and 0.2–0.4 million for VL (https://www.who.int/health-topics/leishmaniasis). Seven countries (Brazil, Ethiopia, India, Kenya, Somalia, South Sudan, and Sudan) report a high VL burden, and 10 countries (Afghanistan, Algeria, Bolivia, Brazil, Colombia, Iran, Iraq, Pakistan, Peru, Syria, and Tunisia) a high CL burden [3,4]. 

In the Mediterranean basin, leishmaniases are neglected diseases that are emerging or re-emerging [5,6]. Algeria belongs to the shortlist of the most affected countries for leishmaniasis, with more than 20,000 cases reported each year, and an incidence of 28.19 cases per 100,000 inhabitants [3]. Zoonotic visceral leishmaniasis (ZVL) is caused by *Leishmania infantum,* with dogs acting as the main reservoir and *Phlebotomus longicuspis* and *P. perniciosus* acting as primary vectors [7]. Historically present mainly in the humid and sub-humid regions of northern Algeria, it has extended from its historical foci of Kabylie (Tizi-Ouzou, Bejaïa) to Blida, Chlef, Medea, and Tipaza foci. The highest number of reported cases occurred in 1998 (310 reported cases); an overall increase recorded from 1994 to 2003 was followed by a decrease during the subsequent decade [8]. In Algeria, cutaneous leishmaniasis (CL) caused by *L. major*, *L. infantum,* and *L. tropica* has a 30-fold higher incidence than the visceral form [8]. Zoonotic cutaneous leishmaniasis (ZCL) is caused by *L. major,* in which the proven vector and reservoir are *Phlebotomus papatasi* and *Psammomys obesus,* respectively [9]. The disease is prevalent in 41 out of Algeria’s 48 districts, spanning the North Saharan fringe, and the arid and semi-arid bioclimatic areas, including Biskra, Bordj Bou Arreridj, Batna, Djelfa, Saida, Sétif, M’sila, and Abadla [9,10]. More recently, a spread of the disease has taken place towards M’sila, Ksar Chellala, Djelfa, and Bou-Saada foci [11], and the Northern part of the Tell Atlas, in the Soummam basin [12]. *Leishmania tropica* causes anthroponotic cutaneous leishmaniasis (ACL), a chronic form with less than 100 cases per year that commonly occurs in sympatry with *L. major* [13,14]. It is restricted to Constantine, Annaba, Ghardaia, and Tipaza [13,15,16]. *Phlebotomus sergenti* is considered the proven vector of *L. tropica,* with humans as the primary reservoir. Nevertheless, some animals like *Massoutiera mzabi* (the Mzab gundi from the family Ctenodactylidae) are additional suspected reservoirs [17,18,19]. Sporadic cutaneous leishmaniasis caused by *L. infantum* was first reported by Sergent in 1923 [20]. The parasitological, epidemiological, and clinical characteristics were individualized by Belazzoug et al. (1985) [21]. Izri and Belazzoug (1993) [22] highlighted the vectorial role of *P. perfiliewi* in Ténès. It is responsible for sporadic cutaneous infections all over the coastal regions in northwestern Algeria (Oran, Tlemcen) [7,8,23] and the Algerian Tell Atlas (Tizi-Ouzou, Bouira, Bord Menail, Tipaza, Blida, and Algiers) [24]. 

Herein, we diagnosed and identified *Leishmania* spp. from suspect CL patients originating from Algeria’s geographical areas. This allowed us to update the geographical distribution of *Leishmania* sp. causing cutaneous infections in Algeria. 

## 2. Materials and Methods

### 2.1. Samples and Clinic

The investigation was conducted from Jun 2016 to November 2017 on patients with symptoms reminiscent of cutaneous leishmaniasis, referred to the Hadjout, Biskra, and Saida health centers. The personal information and lesion type (wet or dry), number, location, duration, and travel history were recorded for each patient. Cutaneous biopsies, sampled according to Evans’s protocol [25], were smeared on a microscopic slide, air-dried, fixed with absolute methanol, stained by Giemsa 10% (Sigma-Aldrich, Saint Louis, MO, USA), and directly examined under a light microscope at 500× or 1000× magnification.

### 2.2. Molecular Diagnosis and Typing

The DNA from stained slides was extracted using a Qiagen DNA mini-kit (Hilden, Germany) and precipitated by ethanol [26]. A conventional polymerase chain reaction (PCR) that amplifies a 300–350 bp fragment (depending on the species) of the internal transcribed spacer 1 (ITS1) was performed using LITSR (forward: 5′-CTGGATCATTTTCCGATG-3′) and L5.8S (reverse: 5′-TGATACCACTTATCGCACTT-3′) primers [27]. Negative (absence of target DNA) and positive (presence of DNA from reference *Leishmania* strains) controls were used for each PCR batch. Amplicons were analyzed after electrophoresis in a 1.5% agarose gel containing ethidium bromide. Endonuclease digestion was performed following a previously published protocol [27]. Briefly, 10 μL of the PCR product was incubated at 37 °C in a final volume of 30 μL, containing 2 μL of *BsuRI* (*HaeIII*) (Fermentas, Vilnius, Lithuania), 2 μL of 10× buffer, and 16 μL of distilled water. After 4 h, digested fragments were run on a 3% agarose gel containing ethidium bromide. A DNA ladder of 50 bp (Fermentas) was used to identify diagnostic DNA fragments. 

### 2.3. Sequencing and Typing of Leishmania Isolates

*Leishmania* DNA was subjected to conventional PCR targeting ITS1 (partial sequence), 5.8S (complete sequence), and ITS2 (partial sequence), using forward (ITS1F: 5′-GCAGCTGGATCATTTTCC-3′) and reverse (ITS2R4: 5′-ATATGCAGAAGAGAGGAGGC-3′) primers with an expected length of 430 bp [28,29]. Double-distilled water and purified DNA from *L. major, L. tropica,* and *L. infantum* were used as negative and positive controls for each PCR batch. Amplicon quality was analyzed after electrophoresis in a 1.5% agarose gel with ethidium bromide. PCR products were purified using an Invisorb Fragment CleanUp kit (Stratec Molecular, Berlin, Germany) and sequenced using the same primers for PCR amplification. The sequences were compared to homologous sequences collected in the GenBank database and aligned with the Basic Local Alignment Search Tool (BLAST) (www.ncbi.nlm.nih.gov/BLAST). All sequences were identified as *L. major, L. tropica,* or *L. infantum,* based on ≥99% identity with GenBank sequences. The phylogenetic analysis was carried out using MEGA v.6 software. A phylogenetic tree of *Leishmania* species (identified in this study) and GenBank sequences was constructed using neighbor-joining (NJ) with bootstrap values of 1000 replicates. 

## 3. Results

**Figure 1:**
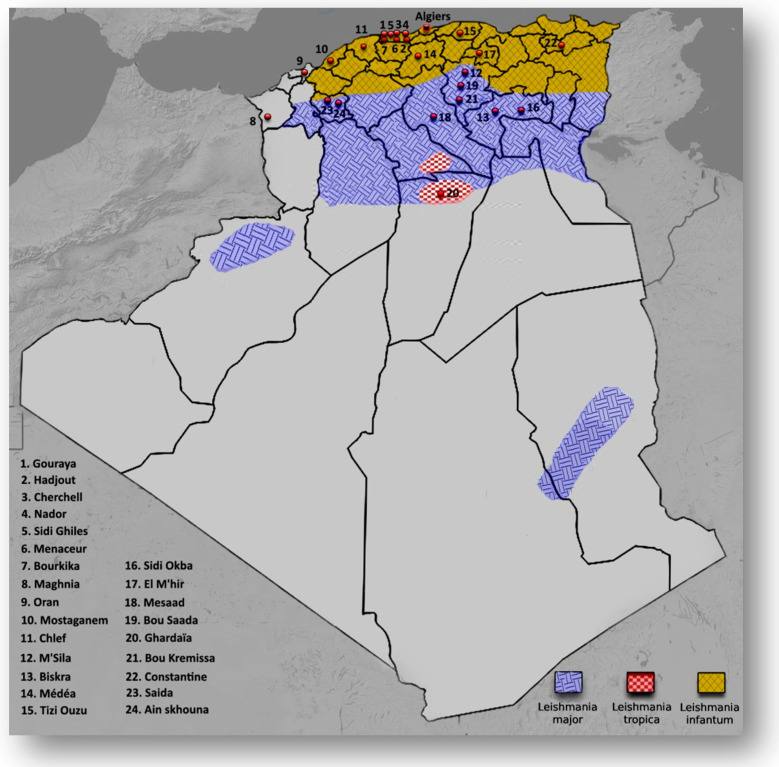
Schematic representation of leishmaniasis endemic regions for *L. major, L. tropica,* and *L. infantum*, and the geographical origin of cutaneous samples processed in the present study (red points).

A total of 32 Giemsa stained smears were prepared from active skin lesions of suspected 27 CL patients referred to the Hadjout, Biskra, and Saida health centers in Algeria (Figure 1). Biopsies were taken from all lesions (one to three lesions) from patients of ages ranging from 3 to 82. After microscopic examination, 27 smears from the 32 lesions processed were positive for *Leishmania* sp. (including four patients with at least two lesions). Five patients were negative for *Leishmania* infection after a microscopic examination. See Table 1 for epidemiological and clinical information of all patients.

**Figure 2:**
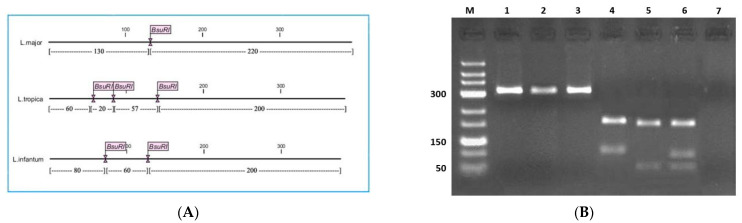
PCR–RFLP of cutaneous biopsies collected in Algeria. (**A**) Schematic representation of *BsuR1* (*HaeIII*) cut sites in amplified fragments of ITS1-rDNA in *Leishmania major*, *L. tropica*, and *L. infantum* (CLC DNA Workbench 5.2 software); (**B**) Ethidium bromide-stained agarose gel of *HaeIII* digested PCR products of *Leishmania* species extracted from Giemsa stained smears. M: molecular marker (50 bp); Lanes 1–3: undigested reference strains of *L. major*, *L. tropica,* and *L. infantum*; Lanes 4–6: digested *L. major*, *L. tropica,* and *L. infantum* isolated from the patients; Lane 7: negative control. For *L. tropica* isolates, the 20 bp fragment could not be observed in an agarose gel electrophoresis. The 57 and 60 bp fragments could not be discriminated; only bands at 200 and 60 bp were indicative and distinguished after agarose gel electrophoresis.

All biopsies were subjected to molecular characterization by PCR–RFLP. A schematic representation of the PCR–RFLP restriction profile is given in Figure 2, along with the restriction profiles generated for selected samples. The twenty-seven smears (24 patients), which were positive after microscopic examination, were also positive for PCR (Table 1). Two lesions, considered as negative after microscopic examination, were positive with PCR. Most lesions caused by *L. major* were located on feet (9/20 cases), whereas lesions due to *L. tropica* were on the head (forehead and face) (Table 1).

**Figure 3:**
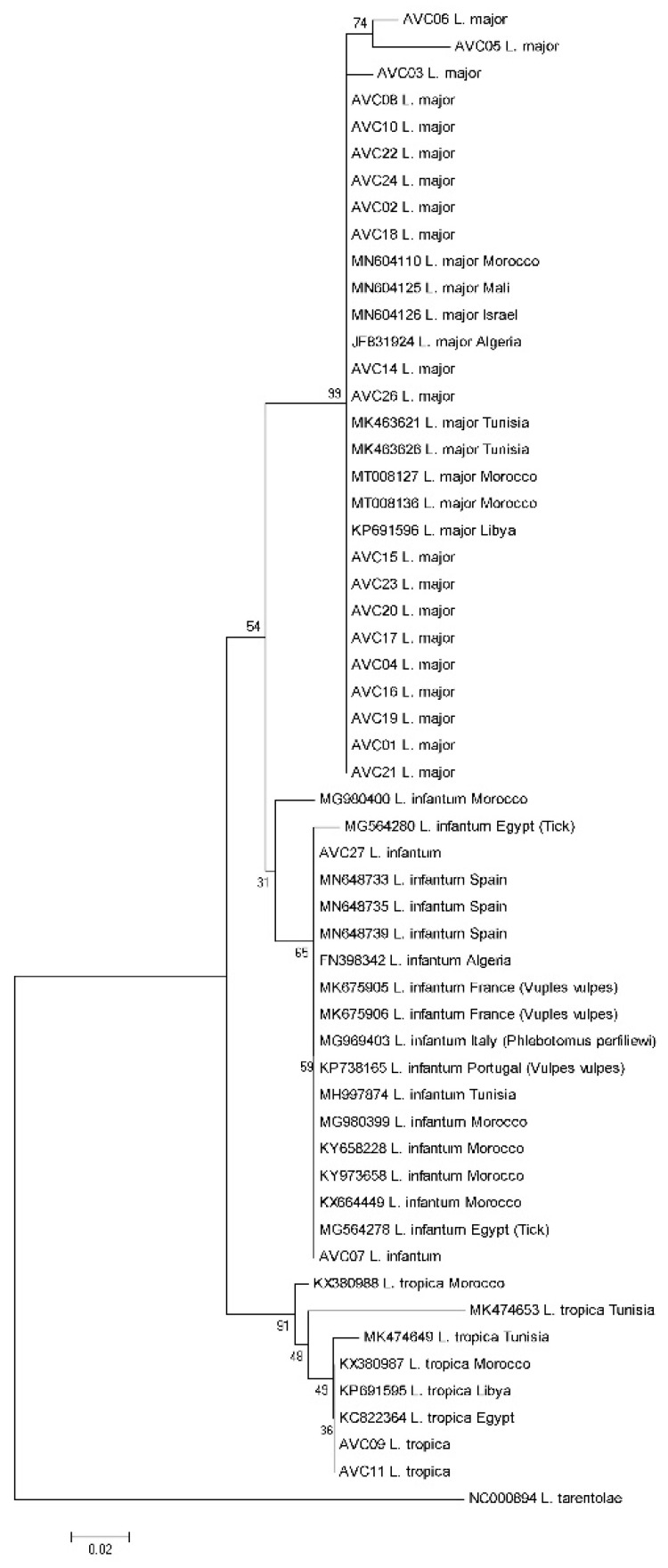
Neighbor-joining (NJ) phylogenetic tree constructed based on ITS1-rDNA sequence of *Leishmania* samples analyzed in the present study (samples entitled AVC) and those collected in GenBank.

The identification of *Leishmania* at the species level was further confirmed by direct sequencing of each isolate’s PCR product. All the sequences were deposited in GenBank under the accession numbers of XN348129 to XN348154. This analysis pinpoints that *Leishmania* sequences from Algerian patients clustered into three well-differentiated and supported clades of *L. major*, *L. tropica,* and *L. infantum* (Figure 3). They gathered with *Leishmania* sequences of various Mediterranean origins collected from GenBank. The two *L. infantum* sequences clustered with *L. infantum* isolated from humans or dogs in different Mediterranean countries, with a bootstrap value of 65% (Figure 3).

## 4. Discussion

The first reported cases of cutaneous and visceral leishmaniases in Algeria date back to 1860 by Hamel, and 1911 by Lemaire [30]. Besides, Edmond and Etienne Sergent and their collaborators were the first, in 1921, to prove sandflies’ vector role. They incriminated the *phlebotomus papatasi* as transmitting the “Clou de Biskra” agent [31,32]. For a long time, *L. major* and *L. infantum* foci were geographically separated in Algeria by the Tell Atlas Mountains, representing a natural barrier. The leishmaniasis epidemiological features seem to be in continuous evolution, resulting in more reports [33]. 

ZCL due to *L. major* is the oldest leishmaniasis, with Biskra in the east and Abadla in the west as the formerly known foci in Algeria [34]. It is prevalent over the entire North-Saharan fringe, corresponding to the arid and semi-arid areas with a progression towards the North. Three CL outbreaks occurred between 2004 and 2006, with 14,822, 25,511, and 14,714 cases, respectively. Besides Biskra and Ababla, Msila experienced an epidemic in 1982, with 8000 recorded cases [35]. In recent years, several new foci of CL due to *L. major*, namely those of El M’hir, Batna, and Bordj Bou Arreridj have emerged on the Northern part of the chain of the Tell Atlas [12,33]. In the present study, in agreement with previously reported cases, we found *L. major* infected patients coming from Ain skhouna, Biskra, El M’hir, Ghardaïa, M’Sila, and Saida [12,13,36,37,38]. Furthermore, we highlight for the first time, the identification of *L. major* in the patients from Bourkika, Bou Kremissa, Bou Saada, Chlef, Hajout, Maghnia, Médéa, Menaceur, Messad, Mostaghanem, Nador, Oran, and Sidi Okba (Figure 1, Table 1). Due to limited information on the medical records of some patients, together with the multiple trips of some of them to ZCL endemic regions, mostly due to seasonal works or vacations, it is quite difficult to justify the precise location of some patients when infected by *Leishmania* parasites (Table 1). Nevertheless, these results confirm the extension of *L. major* in northern Algeria [12]. Studied patients had an age range between 3 to 82 years old, with most lesions located on the feet (45%) (Table 1). Men exhibited the most cutaneous lesions caused by *L. major* (13 out of 20 cases, 65%). Based on the phylogenic tree, we recorded some slight intraspecific heterogeneity for *L. major* (AVC03, AVC05, and AVC06, originating from Biskra, Bou Kremissa, and Bou Saada). Such a genetic diversity has also been reported in other *L. major* endemic regions; Iran [39], Tunisia [40], and Morocco [41]. On the other hand, ZCL has been the subject of multiple studies, mostly isoenzymatic investigations. The characterization of parasites circulating in Algeria using isoenzymatic analysis started in 1981 [42]. Isoenzymatic characterization of *L. major*, the causative agent of zoonotic cutaneous leishmaniasis, evidenced the zymodeme MON-25 in patients, sandfly vectors (*P. papatasi*), and animal reservoirs (*Psammomys* and *Meriones*) [10,43,44,45]. Some years later, a new and less prevalent zymodeme, the MON-269, was identified. It differs from MON-25 by the PGD (phosphogluconate dehydrogenase) enzymatic system [45] (Table 2). 

ACL due to *L. tropica* has been reported in the southern part of the country, particularly in the Oasis of Ghardaia [13]. The MON-301 and MON-306 zymodemes of *Leishmania tropica* are restricted to Constantine [15], Ghardaï [10], and Tipaza [16]. They present some intriguing characteristics, like their inherent lower susceptibility towards antimonial-containing drugs [8,46], or the physiopathological alteration recorded in murine infection models [47]. In the present study, we identified two *L. tropica* cases from Ghardaia and Constantine, which grouped in the same clade with other *L. tropica* sequences from other Mediterranean countries.

Since the discovery of VL’s first case in 1911, the Kabylie has been known for many years as an active focus of the visceral form in particular. Located in the north of the country, it presents a very large geodiversity, with very contrasting portions, both from a bioclimatic, geomorphological, and vegetation point of view, thus offering very diverse biotopes for the different species of sand flies and animal reservoirs. For many years, the highest number of VL cases registered in Algeria occurred in the region of Tizi ouzou (Kabylie). In the recent years, an extension of VL from the old foci in Kabylie (Tizi-Ouzou, Bejaı¨a) to the center (Blida, Chlef, Medea, Tipaza) and the north-eastern part of northern Algeria, with scattered cases occurring in the West (Oran, Tlemcen) [7] have been recorded. The MON-1 and MON-24 zymodemes of *L. infantum* were responsible for zoonotic visceral leishmaniasis and sporadic cutaneous leishmaniasis [48]. They were the most frequently characterized zymodemes in patients, sand flies vectors, and animal reservoirs [9,10,21,22,49]. Although the isoenzymatic characterization allows *Leishmania* species identification, its complexity and prohibitive costs restrict its use in clinical settings [50]. See Table 2 for a synthetic overview of *Leishmania* zymodemes characterized in Algeria. Although most VL and sporadic CL cases due to *L. infantum* are primarily reported in humid regions in northern Algeria, *L. infantum* infection cases are sporadically reported in arid areas [10]. In Algeria, *L. infantum* is associated with diverse clinical and eco-epidemiological situations that raised genetic diversity concerns. The occurrence of three *L. infantum* populations was recorded in Algeria, with two clades encompassing the isolates belonging to the zymodeme MON-1, and a third one, with mainly zymodeme MON-24 isolated from cutaneous leishmaniasis cases [51]. Occasionally recombination events and a generation of hybrid genotypes between MON-1 and MON-24/80 in Algeria have been suspected [52]. In the present study, we identified two SCL cases caused by *L. infantum* in the patients originating from Tizi Ouzu and Cherchell. Due to the restriction in SCL case numbers processed in the present study, our *L. infantum* sequences clustered tightly with other Mediterranean strains, with no significant heterogeneity (Figure 3). 

Parasitological methods (direct examination and in vitro culture) have several limitations regarding their positivity and sensitivity rate. This poor performance of parasitological methods is related to low parasitic load or irregular distribution of amastigotes in lesions [53]. The use of DNA amplification by PCR has allowed *Leishmania* parasites to be identified, and clarified the taxa’s distribution [4,33,54]. In analyzing the phylogenic tree generated with specimens isolated from Algerian patients, we recorded a high level of genetic homogeneity in the isolates of *L. major*, *L. tropica*, and *L. infantum,* which cluster with their counterparts identified in various Mediterranean basin areas (Figure 3). This confirms the identification performed using PCR–RFLP and agrees with *Leishmania* genotyping carried out by Gherbi et al. [55], El Baidouri et al. [56], and Schonian et al. [57] using multilocus microsatellite typing (MLMT) on North African specimens.

**Table 2:** *Leishmania* zymodemes reported in human, sand fly, and animal reservoirs in Algeria.

Clinico-Epidemiological Form	Zymodemes			Reference
	Human	Vector	Reservoir	
ZCL	MON-25 (*L. major*) MON-269 (*L. major*)	MON-25 (*P. papatasi*) MON-269 (*P. papatasi*)	MON-25 (*Psammomys obesus*) MON-269 (*Psammomys obesus*, *Meriones shawi*)	[9,10,45,58,59]
ACL	MON-301 (*L. tropica*) MON-306 (*L. tropica*)	-	-	[13,45,60]
SCL	MON-1 * (*L. infantum*) MON-24 * (*L. infantum*) MON-80 * (*L. infantum*)	MON-24 * (*P. perfilliewi*)	-	[22,61]
ZVL	MON-1 (*L. infantum*) MON-24 (*L. infantum*) MON-33 (*L. infantum*) MON-34 (*L. infantum*) MON-77 (*L. infantum*) MON-78 (*L. infantum*) MON-80 (*L. infantum*) MON-281 (*L. infantum*)	MON-1 (*P. perniciosus*) MON-24 (*P. perfilliewi*)	MON-1 (*Canis familiaris*, *Canis aureus*) MON-24 (*Canis familiaris*) MON-34 (*Canis familiaris*) MON-77 (*Canis familiaris*) MON-80 (*Canis familiaris*) MON-281 (*Canis familiaris*)	[6,61,62,63,64]

*: causing sporadic cases of cutaneous leishmaniasis.

## Figures and Tables

**Table 1:** Clinical and epidemiological data of patients and *Leishmania* diagnosis.

Patients			Lesions		*Leishmania* Diagnosis		Geographical Location				Explanation
Patient Code	Sex	Age (Year)	Number	Site	Microscopy	PCR–RFLP/Sequencing	City	Elevation (m) *	Rainfall (mm)	Bioclimatic Stage **	Disease or Travel History
AVC1	M	39	1	Neck	+	*+/L. major*	Ain skhouna	995	87	Cold semi-arid	
AVC2	M	30	2	Forearm, hand	+	*+/L. major*	Biskra	121	128	Arid (Desertic)	
AVC3	M	41	1	Ankle	+	*+/L. major*	Biskra				
AVC4	F	5	1	Face	+	*+/L. major*	Bourkika	203	642	Mediterranean	
AVC5	M	59	1	Ankle	+	*+/L. major*	Bou Kremissa	328	201	Cold semi-arid	
AVC6	F	9	1	Foot	+	*+/L. major*	Bou Saada	48	98	Arid (Desertic)	
AVC7	M	69	1	Forearm	+	*+/L. infantum*	Cherchell	26	108	Mediterranean	
AVC8	M	49	1	Foot	+	*+/L. major*	Chlef	114	394	Mediterranean	Diabetic
AVC9	F	7	1	Forehead	+	*+/L. tropica*	Constantine	694	512	Mediterranean	
AVC10	F	27	2	Foot	+	*+/L. major*	El M’hir	619	329	Mediterranean	
AVC11	F	16	1	Face	+	*+/L. tropica*	Ghardaïa	497	68	Arid (Desertic)	
AVC12	F	70	1	Foot	-	-	Gouraya	670	642	Mediterranean	
AVC13	M	50	1	Forearm	-	-	Hadjout	81	635	Warm temperate	
AVC14	M	29	1	Hand	+	*+/L. major*	Hadjout				
AVC15	M	41	3	Foot, hand	+	*+/L. major*	Hadjout				
AVC16	M	79	1	Ankle	+	*+/L. major*	Maghnia	374	365	Warm temperate	Travel to Morocco
AVC17	F	74	1	Forearm	+	*+/L. major*	Médéa	981	736	Warm temperate	Diabetic, nephrotic disorder
AVC18	M	10	1	Cheek	+	*+/L. major*	Menaceur	321	661	Warm temperate	2 months inhabitation in Biskra
AVC19	M	64	1	Neck	+	*+/L. major*	Mesaad	592	69	Arid (Desertic)	
AVC20	F	4	1	Foot	+	*+/L. major*	Mostaganem	104	347	Cold semi-arid	Travel to M’Sila
AVC21	F	3	2	Cheek, foot	+	*+/L. major*	M’Sila	471	229	Cold semi-arid	
AVC22	M	18	1	Cheek	+	*+/L. major*	Nador	42	313	Semi-arid	Travel to Biskra
AVC23	M	82	1	Neck	-	*+/L. major*	Oran	0.9	370	Warm temperate	Travel to Tunisia
AVC24	M	28	1	Forearm	+	*+/L. major*	Saida	830	341	Cold semi-arid	
AVC25	F	36	1	Cheek	-	-	Sidi Ghiles	30	634	Mediterranean	
AVC26	F	14	1	Hand	-	*+/L. major*	Sidi Okba	54	127	Arid (Desertic)	
AVC27	M	11	1	Foot	+	*+/L. infantum*	Tizi Ouzu	200	705	Mediterranean	

M: Male; F: Female; *: meter above sea level; **: Based on Köppen climate classification Csa.

## Data Availability

All data are available in the manuscript.

## References

[1] Akhoundi M., Kuhls K., Cannet A., Votýpka J., Marty P., Delaunay P., Sereno D. (2016). A Historical Overview of the Classification, Evolution, and Dispersion of Leishmania Parasites and Sandflies. PLoS Negl. Trop. Dis..

[2] World Health Organization (2018). https://www.who.int/health-topics/leishmaniasis#tab=tab_1.

[3] Alvar J., Vélez I.D., Bern C., Herrero M., Desjeux P., Cano J., Jannin J., den Boer M. (2012). Leishmaniasis worldwide and global estimates of its incidence. PLoS ONE.

[4] Akhoundi M., Downing T., Votýpka J., Kuhls K., Lukeš J., Cannet A., Ravel C., Marty P., Delaunay P., Kasbari M. (2017). *Leishmania* infections: Molecular targets and diagnosis. Mol. Asp. Med..

[5] Kholoud K., Bounoua L., Sereno D., El Hidan M., Messouli M. (2020). Emerging and Re-Emerging Leishmaniases in the Mediterranean Area: What Can Be Learned from a Retrospective Review Analysis of the Situation in Morocco during 1990 to 2010?. Microorganisms.

[6] Chaara D., Haouas N., Dedet J.P., Babba H., Pratlong F. (2014). Leishmaniases in Maghreb: An endemic neglected disease. Acta Trop..

[7] Adel A., Boughoufalah A., Saegerman C., De Deken R., Bouchene Z., Soukehal A., Berkvens D., Boelaert M. (2014). Epidemiology of Visceral Leishmaniasis in Algeria: An Update. PLoS ONE.

[8] Eddaikra N., Ait-Oudhia K., Kherrachi I., Oury B., Moulti-Mati F., Benikhlef R., Harrat Z., Sereno D. (2018). Antimony susceptibility of *Leishmania* isolates collected over a 30-year period in Algeria. PLoS Negl. Trop. Dis..

[9] Belazzoug S. (1983). The new focus of cutaneous leishmaniasis of M’sila (Algeria). Natural infection cf Psammomys obesus (Rodentia, Gerbillidae). Bull. Soc. Pathol. Exot. Filiales..

[10] Harrat Z., Pratlong F., Belazzoug S., Dereure J., Deniau M., Rioux J.A., Belkaid M., Dedet J.P. (1996). *Leishmania infantum* and *L. major* in Algeria. Trans. R. Soc. Trop. Med. Hyg..

[11] Kabbout N., Merzoug D., Chenchouni H. (2016). Ecological Status of Phlebotomine Sand flies (Diptera: Psychodidae) in Rural Communities of North-eastern Algeria. J. Arthropod Borne Dis..

[12] Boudrissa A., Cherif K., Kherrachi I., Benbetka S., Bouiba L., Boubidi S.C., Benikhlef R., Arrar L., Hamrioui B., Harrat Z. (2012). Extension de Leishmania major au nord de l’Algérie. Spread of *Leishmania major* to the north of Algeria. Bull. Soc. Pathol. Exot..

[13] Harrat Z., Boubidi S.C., Pratlong F., Benikhlef R., Selt B., Dedet J.P., Ravel C., Belkaid M. (2009). Description of Leishmania close to *Leishmania killicki* (Rioux, Lanotte et Pratlong, 1986) in Algeria. Trans. Roy. Soc. Trop. Med. Hyg..

[14] Garni R., Tran A., Guis H., Baldet T., Benallal K., Boubibi S., Harrat Z. (2014). Remote sensing, land cover changes, and vector-borne diseases: Use of high spatial resolution satellite imagery to map the risk of occurrence of cutaneous leishmaniasis in Ghardaïa, Algeria. Infect. Genet. Evol..

[15] Mihoubi I., Picot S., Hafirassou N., de Monbrison F. (2008). Cutaneous leishmaniasis caused by *Leishmania tropica* in Algeria. Trans. R. Soc. Trop. Med. Hyg..

[16] Izri A., Bendjaballah A., Andriantsoanirina V., Durand R. (2014). Cutaneous leishmaniasis caused by *Leishmania killicki*, Algeria. Emerg. Infect. Dis..

[17] Jaouadi K., Haouas N., Chaara D., Gorcii M., Chargui N., Augot D., Pratlong F., Dedet J.-P., Ettlijani S., Mezhoud H. (2011). First detection of *Leishmania killicki* (*Kinetoplastida*, *Trypanosomatidae*) in *Ctenodactylus gundi* (*Rodentia*, *Ctnenodactilidae*), a possible reservoir of human cutaneous leishmaniasis in Tunisia. Parasit Vectors.

[18] Boubidi S.C., Benallal K., Boudrissa A., Bouiba L., Bouchareb B., Garni R., Bouratbine A., Ravel C., Dvorak V., Votypka J. (2011). Phlebotomus sergenti (Parrot, 1917) identified as Leishmania killicki host in Ghardaïa, south Algeria. Microbes Infect..

[19] Maroli M., Feliciangeli M.D., Bichaud L., Charrel R.N., Gradoni L. (2013). Phlebotomine sandflies and the spreading of leishmaniases and other diseases of public health concern. Med. Vet. Entomol..

[20] Sergent E., Gueidon E. (1923). Chronique duboutond’Orient en Algérie. «Le clou de Mila». Arch. Institut. Pasteur Algerie.

[21] Belazzoug S., Lanotte G., Maazoun R., Pratlong F., Rioux J.A. (1985). Un nouveau variant enzymatique de Leishmania infantum Nicolle, 1908, agent de la leishmaniose cutanée du Nord de l’Algérie. Ann. Parasitol. Hum. Comp..

[22] Izri M.A., Belazzoug S. (1993). *Phlebotomus (Larroussius) perfiliewi* naturally infected with dermotropic *Leishmania infantum* at Tenes, Algeria. Trans. R. Soc. Trop. Med. Hyg..

[23] Addadi K., Dedet J. (1976). Epidemiology of leishmaniasis in Algeria. 6- Survey of clinical cases of infantile visceral leishmaniasis from 1965 to 1974. Bull. Soc. Path. Exot..

[24] Barchiche A.N., Madiou M. (2009). Recrudescence des leishmanioses cutanées: À propos de 213 cas dans la wilaya de Tizi-Ouzou. Outbreak of cutaneous leishmaniasis: About 213 cases in the province of Tizi-Ouzou. Pathol. Biol..

[25] Evans D. (1989). UNDP/WORLD BANK/WHO. Handbook on Isolation, Characterization and Cryopreservation of Leishmania.

[26] Kelly J.M., Hyde J.E. (1993). Method in Molecular Biology. Isolation of RNA & DNA from Leishmania.

[27] Schönian G., Nasereddin A., Dinse N., Schweynoh C., Schalling H.D., Presbe W., Jaffe C. (2003). PCR diagnosis and characterization of *Leishmania* in local and imported clinical samples. Diag. Microbiol. Infect..

[28] Akhoundi M., Hajjaran H., Baghaei A., Mohebali M. (2013). Geographical distribution of leishmania species of human cutaneous leishmaniasis in fars province, southern iran. Iran J. Parasitol..

[29] Akhoundi M., Mohebali M., Asadi M., Mahmodi M.R., Amraei K., Mirzaei A. (2013). Molecular characterization of Leishmania spp. in reservoir hosts in endemic foci of zoonotic cutaneous leishmaniasis in Iran. Folia Parasitologica.

[30] Sergent E., Parrot L. (1926). Chronique du Bouton d’Orient. Arch. Inst. Pasteur Alger..

[31] Sergent E., Parrot I., Donatien A., Beguet M. (1921). Transmission du clou de Biskra par le phlébotome Phlebotomus papatasi (Scop). CR Acad. Sci..

[32] Louzir H., Aoun K., Späth G.F., Laouini D., Prina E., Victoir K., Bouratbine A. (2013). Leishmania epidemiology, diagnosis, chemotherapy and vaccination approaches in the international network of Pasteur Institutes. Med. Sci..

[33] Bachi F., Icheboudene K., Benzitouni A., Taharboucht Z., Zemmouri M. (2019). Epidemiology of Cutaneous Leishmaniasis in Algeria through Molecular Characterization. Bull. Soc. Pathol. Exot..

[34] Hamel H. (1860). Etude comparée des boutons d’Alep et de Biskra. Rec. Mém. Med.Chir. Pharm. Milit..

[35] Elhadj H., Kerboua Ziari Y.S., Selmane S. (2015). Cutaneous Leishmaniasis Modeling: The case of Msila Province in Algeria. Int. J. Innov. Appl. Stud..

[36] Bennai K. (2019). Surveillance et Contrôle des Leishmanioses dans le nord de l’Algérie. Ph.D. Thesis.

[37] Cherif K., Boudrissa A., Cherif M.H., Harrat Z. (2012). A social program for the control of zoonotic cutaneous leishmaniasis in M’Sila, Algeria. Sante Publique.

[38] Bounoua L., Kahime K., Houti L., Blakey T., Ebi K.L., Zhang P., Imhoff M.L., Thome K.J., Dudek C., Sahabi S.A. (2013). Linking climate to incidence of zoonotic cutaneous leishmaniasis (L. major) in pre-Saharan North Africa. Int. J. Environ. Res. Public Health.

[39] Tashakori M., Kuhls K., Al-Jawabreh A., Mauricio I.L., Schönian G., Farajnia S., Alimohammadian M.H. (2006). Leishmania major: Genetic heterogeneity of Iranian isolates by single-strand conformation polymorphism and sequence analysis of ribosomal DNA internal transcribed spacer. Acta Trop..

[40] Attia H., Sghaier R.M., Gelanew T., Bali A., Schweynoch C., Guerfali F.Z., Mkannez G., Chlif S., Belhaj-Hamid N., Dellagia K. (2016). Genetic micro-heterogeneity of *Leishmania major* in emerging foci of zoonotic cutaneous leishmaniasis in Tunisia. Infect. Gen. Evol..

[41] Ait Kbaich M., Mhaidi I., Daoui O., Ait Maatallah I., Riyad M., Akarid K., Lemrani M. (2020). Population structure of *leishmania major* in southeastern morocco. Acta Trop..

[42] Lanotte G., Rioux J.A., Maazoun R., Pasteur N., Pratlong F., Lepart J. (1981). Application de la method numérique à la taxonomie du genre Leishmania Ross,1903. Apropos de 146 souches originaires de l’Ancien Monde Utilisation des allozymes. Corollaires épidémiologiques et phylétiques. Ann. Parasit. Hum. Comp..

[43] Rioux J.A., Guilvard E., Dereure J., Lanotte G., Denial M., Pratlong F., Serres E., Belmonte A., Rioux J.A. (1986). Infestation naturelle de Phlebotomus papatasi (Scopoli, 1786), par Leishmania major MON-25. A propos de 28 souches isolées dans un foyer du Sud marocain. Leishmania. Taxonomie et Phylogenèse. Applications Éco-Épidémiologiques.

[44] Maazoun R., Pratlong F., Lanotte G., Rioux J.A., Rioux J.A. (1986). Le complexe Leishmania major. A propos de l’analyse numérique de 35 souches identifiées par la méthode enzymatique. Leishmania. Taxonomie et phylogenèse. Applications éco-épidémiologiques.

[45] Pratlong F., Dereure J., Ravel C., Lami P., Balard Y., Serres G., Lanotte G., Rioux J.A., Dedet J.P. (2009). Geographical distribution and epidemiological features of Old World cutaneous leishmaniasis foci, based on the isoenzyme analysis of 1048 strains. Trop. Med. Int. Health.

[46] Sereno D., Harrat Z., Eddaikra N. (2019). Meta-analysis and discussion on challenges to translate Leishmania drug resistance phenotyping into the clinic. Acta Trop..

[47] Eddaikra N., Kherachi Djenad I., Benbetka S., Benikhlef R., Aït-Oudhia K., Moulti-Mati F., Oury B., Sereno D., Harrat Z. (2016). Development of a Murine Infection Model with Leishmania killicki, Responsible for Cutaneous Leishmaniosis in Algeria: Application in Pharmacology. BioMed Res. Int..

[48] Marty P., Lacour J.P., Pratlong F., Perrin C., Del Giudice P., Le Fichoux Y. (1998). Leishmaniose cutanée localisée due à Leishmania infantum MON-1 contractée dans le Nord de l’Algérie. Bull. Soc. Pathol. Exot..

[49] Dedet J.P., Belazzoug S., Chang K.P., Bray R.S. (1985). Leishmaniasis in North Africa.

[50] Guerbouj S., Mkada–Driss I., Guizani I. (2014). Molecular Tools for Understanding Eco-Epidemiology, Diversity and Pathogenesis of Leishmania Parasites. Leishmaniasis—Trends in Epidemiology, Diagnosis and Treatment.

[51] Seridi N., Amroc A., Kuhls K., Belkaid M., Zidane C., Al-Jawabreh A., Schonian G. (2008). Genetic polymorphism of Algerian *Leishmania infantum* strains revealed by multilocus microsatellite analysis. Microb. Infec..

[52] Chargui N., Amro A., Haouas N., Schönian G., Babba H., Schmidt S., Ravel C., Lefebvre M., Bastien P., Chaker E. (2009). Population structure of Tunisian Leishmania infantum and evidence for the existence of hybrids and gene flow between genetically different populations. Int. J. Parasitol..

[53] Mouttaki T., Morales-Yuste M., Merino-Espinosa G., Chiheb S., Fellah H., Martin-Sanchez J., Riyad M. (2014). Molecular diagnosis of cutaneous leishmaniasis and identification of the causative Leishmania species in Morocco by using three PCR-based assays. Parasit Vectors.

[54] Mirzaei A., Ahmadipour F., Cannet A., Marty P., Delaunay P., Perrin P., Dorkeld F., Sereno D., Akhoundi M. (2018). Immunodetection and molecular determination of visceral and cutaneous Leishmania infection using patients’ urine. Infect. Genet. Evol..

[55] Gherbi R., Bounechada M., Latrofa M.S., Annoscia G., Tarallo V.D., Dantas-Torres F., Otranto D. (2020). Phlebotomine sand flies and Leishmania species in a focus of cutaneous leishmaniasis in Algeria. PLoS Negl. Trop. Dis..

[56] El Baidouri F., Diancourt L., Berry V., Chevenet F., Pratlong F., Marty P., Ravel C. (2013). Genetic Structure and Evolution of the *Leishmania* Genus in Africa and Eurasia: What Does MLSA Tell Us. PLoS Negl. Trop. Dis..

[57] Schönian G., Kuhls K., Mauricio I.L. (2011). Molecular approaches for a better understanding of the epidemiology and population genetics of *Leishmania*. Parasitology.

[58] Izri M.A., Belazzoug S., Pratlong F., Rioux J.A. (1992). Isolement de L. major chez Phlebotomus papatasi à Biskra (Algérie). Fin d’une épopée éco-épidémiologique. Ann. Parasitol. Hum. Comp..

[59] Belazzoug S. (1986). Découverte d’un Meriones shawi (Rongeur, Gerbillidé) naturellement infesté par Leishmania dans le nouveau foyer de Ksar Chellal (Algérie). Bull. Soc. Pathol. Exot..

[60] Mansouri R., Pratlong F., Bachi F., Hamrioui B., Dedet J.P. (2012). The first isoenzymatic characterizations of the Leishmania strains responsible for cutaneous leishmaniasis in the Area of Annaba (Eastern Algeria). Open Conf. Proc. J..

[61] Benikhlef R., Harrat Z., Toudjine M., Djerbouh A., Bendali-Braham S., Belkaid M. (2004). Présence de Leishmania infantum MON-24 chez le chien. Med. Trop..

[62] Ait-Oudhia K., Lami P., Lesceu S., Harrat Z., Hamrioui B., Dedet J.P., Pratlong F. (2009). Increase in the prevalence of canine leishmaniasis in urban Algiers (Algeria) following the 2003 earthquake. Ann. Trop. Med. Parasitol..

[63] Harrat Z., Belkaid M. (2003). Leishmaniasis in Algiers: Epidemiologic data. Bull. Soc. Pathol. Exot..

[64] Harrat Z., Addadi K., Belkaid M., Tabet-Derraz O. (1992). Visceral leishmaniasis in Algeria. Cases reported of visceral leishmaniasis (1985–1990). Bull. Soc. Pathol. Exot..

